# Isoform-specific anti-MeCP2 antibodies confirm that expression of the e1 isoform strongly predominates in the brain

**DOI:** 10.12688/f1000research.2-204.v1

**Published:** 2013-10-04

**Authors:** Lara Kaddoum, Nicolas Panayotis, Honoré Mazarguil, Giuseppina Giglia-Mari, Jean Christophe Roux, Etienne Joly

**Affiliations:** 1 CNRS, Institut de Pharmacologie et de Biologie Structurale, Toulouse, F-31077, France; 2 Université de Toulouse, UPS, Institut de Pharmacologie et de Biologie Structurale, Toulouse, F-31077, France; 3 INSERM U910, Unité de Génétique Médicale et Génomique Fonctionnelle, Faculté de Médecine de La Timone, Marseille, F-13385, France; 4 Aix-Marseille Université, Faculté de Médecine de La Timone, Marseille, F-13385, France; 5 Department of Biological Chemistry, Weizmann Institute of Science, Rehovot, 76100, Israel

## Abstract

Rett syndrome is a neurological disorder caused by mutations in the *MECP2* gene.  MeCP2 transcripts are alternatively spliced to generate two protein isoforms (MeCP2_e1 and MeCP2_e2) that differ at their N-termini. Whilst mRNAs for both forms are expressed ubiquitously, the one for *MeCP2_e1* is more abundant than for *MeCP2_e2* in the central nervous system. In transfected cells, both protein isoforms are nuclear and colocalize with densely methylated heterochromatic foci. With a view to understanding the physiological contribution of each isoform, and their respective roles in the pathogenesis of Rett syndrome, we set out to generate isoform-specific anti-MeCP2 antibodies. To this end, we immunized rabbits against the peptides corresponding to the short amino-terminal portions that are different between the two isoforms. The polyclonal antibodies thus obtained specifically detected their respective isoforms of MeCP2 in Neuro2a (N2A) cells transfected to express either form. Both antisera showed comparable sensitivities when used for Western blot or immunofluorescence, and were highly specific for their respective isoform. When those antibodies were used on mouse tissues, specific signals were easily detected for Mecp2_e1, whilst Mecp2_e2 was very difficult to detect by Western blot, and even more so by immunofluorescence. Our results thus suggest that brain cells express low amounts of the Mecp2-e2 isoform. Our findings are compatible with recent reports showing that MeCP2_e2 is dispensable for healthy brain function, and that it may be involved in the regulation of neuronal apoptosis and embryonic development.

## Introduction

Rett syndrome (RTT) is a dominant X-linked neurological disorder that affects girls. It is a progressive disease with symptoms appearing around 6 to 18 months after birth. After a normal developmental period, girls show growth retardation, microcephaly, stereotypic hand movements, motor abnormalities, mental retardation and communication dysfunction ^1^. Most RTT cases are sporadic, but using information from rare familial cases, Amir *et al.* did manage to identify mutations in the *MECP2* gene as the origin of 95% of classic RTT cases ^2^.

The *MECP2* gene encodes for the methyl-CpG binding protein 2, an abundant nuclear protein identified in 1992 for its capacity to bind methylated DNA ^3^. MeCP2 is particularly abundant in mature neurons and favors brain development and maturation ^4– 6^. Although MeCP2 was initially thought to be mostly a transcription factor, it has become apparent, over the past few years, that MeCP2 is expressed at extremely high levels in mature neurons, and that one of its central functions is to influence chromatin architecture by assuming a histone H1-like role ^7^.

**Figure 1.**
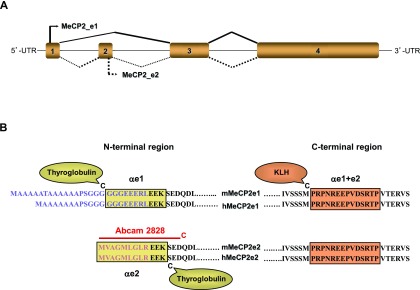
A). Graphical representation of the *MECP2* gene and mRNA. Adaptation from Mnatzakanian *et al.* ^9^ and Kriaucionis and Bird ^10^, showing alternative splicing for the e1 (or B, or α) isoform above the gene and for the e2 (or A or β) isoform below the gene. **B**). Alignment of mouse (m) and human (h) MeCP2_e1 and MeCP2_e2 proteins sequences. Boxes delineate the peptides’ sequences chosen for immunization: in the N-terminal region peptides correspond to regions that differ between the two isoforms, whereas in the C-terminal region, the amino acid sequences are identical to both isoforms. The sequence of the 15-mer peptide used to produce the Abcam2828 antibody is indicated by the red line.

The *MECP2* gene consists of four exons giving rise to two different isoforms of the protein due to an alternative splicing of the mRNA. In addition, the *MECP2* mRNA has a long highly conserved 3′-UTR with three sites of polyadenylation generating three different transcripts for each isoform. The first isoform to be described was MeCP2_e2, which contains all four exons with the initiation site in exon 2 giving rise to a protein of 486 amino acids in humans and 484 in mice ^8^. MeCP2_e2 is also sometimes referred to as MeCP2A, mostly in humans, or MeCP2β, mostly in mice. The MeCP2_e1 isoform (also called MeCP2B or MeCP2α was identified eleven years later, both in human ^9^ and mouse ^10^. It lacks exon 2, and thus consists of exons 1, 3 and 4, with the starting codon in exon 1, giving rise to a protein of 498 amino acids in humans, and 501 in mice (see Bienvenu and Chelly ^8^ and Figure 1A).

RT-PCR analyses have revealed the presence of two transcripts in all tissues in human and mouse, and also demonstrated that the *MeCP2_e1* mRNA isoform is more abundant than *MeCP2_e2* in the brain, thymus and lung ^5, 9– 11^. Although mRNAs for MeCP2 containing exon 2 have been identified in many placental mammals, including primates, carnivores and herbivores, many arguments suggest that the MeCP2_e1 protein may be the dominant form expressed in the brain, and the one which is more relevant to the physiopathology of RTT.

Firstly, in the *MeCP2_e2* mRNA, the ATG start codon present in exon 1 is followed by a very short open reading frame that terminates after 55 nucleotides (in mouse) before the starting codon in exon 2. Kriaucionis and Bird actually demonstrated that the presence of this first ATG results in very inefficient translation of the MeCP2_e2 protein ^10^.

Second, the ancestral form of MeCP2 inferred from sequence comparisons with non-mammalian vertebrates corresponds to MeCP2_e1 ^10^.

Third, until now, in the hundreds of sequences for *MeCP2* genes obtained from patients affected by RTT, no mutation has yet been found in exon 2. On the other hand, work carried out between 2005 and 2009 has already revealed the presence of more than 10 different mutations (deletions and missense) in exon 1 in patients with classical or atypical (mild and severe form) RTT ^12^.

Fourth, in two patients showing classical phenotypes of RTT, but without seizures or microcephaly, Saunders *et al.* identified mutations affecting the initiation codon of *MeCP2_e1* ^12^, which would result in the lack of translation of the MeCP2_e1 protein, but would be expected to somewhat ‘restore’ higher expression of MeCP2_e2, similarly to what has been reported for the mouse cDNA ^10^. Furthermore, at the meeting of the American Society for Human Genetics held in San Francisco in November 2012, the group of J. LaSalle reported that transgenic KO mice carrying the equivalent single point mutation in exon 1, invalidating the initiation codon for MeCP2-e1, recapitulated a typical RTT-like phenotype ^13^.

Lastly, a recent report documented that, in four classical RTT patients harbouring mutations in exon 1, there were normal levels of MeCP2_e2 mRNA ^14^.

On the other hand, even if the mRNA coding for the MeCP2_e2 protein isoform does not suffice to prevent a RTT phenotype ^15^, this isoform must be able to fulfill most functions of MeCP2_e1 since, in MeCP2 KO mice, the pathologic phenotype could be rescued by a tau-driven transgene based on the MeCP2_e2 cDNA ^16^. It is also worthy of note that for historical reasons, many of the studies based on the expression of recombinant MeCP2 were using cDNA constructs coding for the MeCP2_e2 isoform. A recent study has, however, established that expression of either isoform could prevent the RTT-like phenotype in MeCP2 ^-/y^ mice, although the rescue of the clasping and motor phenotypes was significantly higher with MeCP2_e1 ^17^.

To explore the expression of each protein isoform *in vivo*, and understand their respective contributions to Mecp2-related pathologies and especially RTT, we thought that it would be very valuable to have access to antibodies that could detect them specifically and separately. In this regard, however, all the anti-MeCP2 antibodies that we had at our disposal had been raised against portions common to the two isoforms, and would thus recognize them both. With a view to obtain truly isoform-specific antibodies, and since the commonly accepted minimal size for a peptide epitope is five amino acids, we chose to immunize rabbits with shorter peptides that overlapped by only three amino acids between the two MeCP2 isoforms. Since the initiation of our study, we have been made aware of the existence of commercially available polyclonal rabbit antibodies generated against the first 15 amino acids of *MeCP2_e2*, namely Abcam2828, and Thermo PA1-881. For our purpose, however, a concern with these antibodies is that the 15 amino acid sequence of the peptides used for immunizations overlaps with the *MeCP2_e1* sequence by six amino acids, and the possibility thus remains that this could be sufficient to lead to some cross-reaction against e1.

Here, we describe the generation of these two isoform-specific antibodies, and their characterization in comparison to Abcam2828 by staining of cells transfected to express either one or the other of the two isoforms, both by immunofluorescence and Western blotting. Those antibodies were then used to compare expression of the two isoforms in the brain, which revealed a prominent expression of the e1 isoform, whilst e2 remained barely detectable.

## Material and methods

### Ethical statement

All experimental procedures involving animals were carried out in keeping with the European guidelines for the care and use of laboratory animals (EU directive 2010/63/EU). No specific approval was required for this study since animals were bred and sacrificed under approved conditions, and not submitted to any experimental procedures. Both Jean Christophe Roux in Marseille and Etienne Joly hold licenses to experiment on animals, and to oversee other people doing experiments under their supervision and responsibility. Their respective numbers are 13–405 for Dr Roux, and 31–40 for Etienne Joly.

### Rabbit immunization

For each antibody, two New Zealand white rabbits were immunized with a synthetic peptide corresponding either to amino acids 1–12 of human MeCP2_e2 (MVAGMLGLREEK-C), or to amino acids 14–24 of human MeCP2_e1 (C-GGGEEERLEEK) ( Figure 1). The cysteine residues were added for conjugation to carrier proteins for immunizations, or to resins for affinity purification. For immunization, we chose thyroglobulin, to which each peptide was coupled using maleimide. Rabbit immunizations were carried out by Eurogentec, Angers, France. Four injections with the thyroglobulin-conjugated peptides were performed on day 0, 21, 49, and 77 (two rabbits per peptide). With the e2 peptide, the sera obtained from both rabbits gave indistinguishable results in all immunofluorescence and Western blot experiments where they were initially compared, and most subsequent experiments were thus carried out with only one of the two. For the e1 peptide, however, only one rabbit responded satisfactorily. The sera from the three rabbits having responded satisfactorily were affinity-purified by MilleGen (Labège, France) on columns coupled to the appropriate peptides. We will refer to those antibodies as αe1 and αe2 respectively.

The third antibody, αe1+e2, recognizes both MeCP2 isoforms, and was raised against (C-PRPNREEPVDSRTP) as described previously by others ^18^ and ourselves ^19^. This polyclonal antibody was produced for us by MilleGen (Labège, France). Rabbits received five injections on day 0, 12, 23, 44 and 57 with the KLH-conjugated peptide we provided. One of the two rabbits responded satisfactorily, and the serum obtained from that rabbit gave sufficient titers and specificity to be used without further purification.

All three peptides used were synthesized by FMOC chemistry, purified by HPLC and coupled to carrier proteins by the IPBS core facility. Thyroglobulin, KLH and maleimide were all purchased from Sigma.

### Abcam antibody 2828

This antibody was purchased from Abcam (Cambridge, UK), kept as frozen aliquots as recommended by the manufacturer, and used in parallel to the three antibodies we generated ourselves.

### Mice

Mice used in this study were male B6.129P2(C)-Mecp2tm1.1Bird/J obtained from the Jackson Laboratory (USA), carrying either the inactivated *Mecp2* allele, or their wild type (WT) littermates. For the mouse experiments, hemizygous mutant males ( *Mecp2* ^-/y^) were generated by crossing heterozygous females ( *Mecp2* ^+/-^) with C57BL/6 males. Genotyping was performed by routine PCR technique according to the Jackson Laboratory protocols as previously described ^20^. A total number of 10 Mecp2−/y and 10 WT mice were used in the study. For histology, the Mecp2−/y and WT mice were sacrificed at postnatal day 24 (P24; n=2 WT; n=2 Mecp2−/y) and day 55 (P55; n=4 WT; n=4 Mecp2−/y). For Western blotting, a total of eight mice (four pairs of WT and Mecp2−/y littermates) were sacrificed over the course of the study, at ages ranging from 3 to 6 weeks.

### Cell culture and transfections

pcDNA3.1(A) vectors expressing Myc-tagged human MeCP2_e1 and MeCP2_e2 were provided by Dr. Berge A. Minassian of the Hospital for Sick Children, Toronto, Ontario, Canada ^9^.

Neuro2a (N2A) cells, a mouse neuroblastoma cell line, were originally obtained from the ATCC (USA), and G418-resistant clones of stably transfected N2A cells expressing Myc-His-tagged human MeCP2_e1 or MeCP2_e2 were maintained in DMEM 10% fetal calf serum supplemented with 100 U/ml penicillin, 100 μg/ml streptomycin, 0.1 mM non-essential amino acids, 1 mM sodium pyruvate (+ 0.5 mg/ml G418 for the transfectants). All tissue culture reagents were from Gibco Life Technologies.

### Immunofluorescence staining of tissue culture cells and microscopy

2.5×10 ^5^ N2A cells were plated overnight on glass coverslips in 24-well plates. Cells were washed twice with PBS and fixed with paraformaldehyde 3.7% in PBS for 30 min at room temperature (RT). After two washes with PBS, cells were permeabilized in 0.3% Triton-X100 for 10 min at RT. After 2×15 min washes with PBS/FCS 2%, cells were incubated with Rabbit anti-MeCP2 serum (1:300 dilution in PBS/FCS 10%) for 1 h at RT. Cells were again washed 3×5 min before incubation with Alexa Fluor 633-labelled polyclonal goat anti-rabbit, (Invitrogen, cat n° A-21070) diluted 1:300 in PBS/FCS 10% for 1 h at RT. Cells were washed 3×5 min with PBS/FCS 2% and after a final wash with PBS, coverslips were mounted in Vectashield Mounting medium with DAPI (4′,6-diamidino-2-phenylindole, dihydrochloride; Vector Labs, Peterborough, UK). We used a Zeiss LSM710 confocal laser scanning microscope, with a 40× oil immersion objective to visualize the stained cells. Images were acquired and analyzed with the ImageJ software (Version 1.47, http://rsb.info.nih.gov/ij/).

### Western blots (WB)

Cellular extracts from N2A cells or mouse brain tissues were prepared as described previously ^21^. After evaluation of the protein contents using a Bradford assay (BioRad), adjusted amounts of proteins from these extracts were loaded onto acrylamide gels. After PAGE separation, proteins were transferred to nitrocellulose membrane (0.45 μm Whatman) (VWR, France). Membranes were blocked overnight at 4°C with TBS (Tris 10 mM, NaCl 0.15 M, pH 7.4 containing 3% skimmed powder milk (non-fat Régilait, France) and 0.1% Tween-20). TBS blocking buffer was always centrifuged (100000 ***g*** for 45 min) and filtered with 0.2 mm filters. Membranes were then incubated with one of the four rabbit anti-MeCP2 antibodies diluted in blocking buffer for 1 h at RT (αe1 was diluted to 1/4000, αe2 to 1/4000, Abcam2828 to 1/1000 and αe1+e2 to 1/4000). After 4×10 min washes in PBS/0.1% Tween buffer, membranes were incubated with goat anti-rabbit conjugated to horseradish peroxidase (HRP) (BioRad, 1:10000 dilution in blocking buffer) for 1 h at RT. Finally, the blots were washed 4×10 min in PBS/0.1% Tween buffer and revealed with an ECL kit (Pierce, Thermo Fisher Scientific, Brebières, France) and using Amersham hyperfilm ECL (high performance chemiluminescence, GE Healthcare).

### Brain tissue immunostaining

Mice were sacrificed with a lethal pentobarbitone injection (100 mg kg ^−1^ i.p.) and transcardially perfused with NaCl for 1 min, followed by PBS 0.1 M containing 4% paraformaldehyde for 10 min. Dissected brains were then postfixed for 5 h in the same solution, placed overnight in PBS 0.1 M containing 20% sucrose and frozen at −80°C.

Brain sections (20 µm) were cut using a cryostat (Microm Microtech, France), permeabilized for 15 min in PBS Triton 0.1%, blocked for 45 min with 7% normal goat serum (NGS, cat n° 005–000_121, Jackson ImmunoResearch Laboratories, Newmarket, England), and incubated overnight at RT with primary antibodies (αe1, αe2 or Abcam2828) diluted 1/300 in PBS containing 3.5% NGS. The following day, sections were washed, incubated 2 h at RT with goat polyclonal anti-rabbit alexa 546 (Life technologies, cat. n° 11010 diluted 1:400 in PBS containing 3.5% NGS and re-washed. After 5 min incubation with DAPI, slides were mounted in Shandon Immu-Mount (ThermoFisher). Immunostained slices were analyzed using a Leica DMR microscope (Leica Microsystems, Wetzlar, Germany) equipped with a CoolSNAP camera (Princeton Instruments, Trenton, NJ, USA). Pictures were then analyzed with ImageJ software.

Immunoquantification of the staining levels was performed using the same protocol previously described in detail ^22^. In order to compare the intensity of staining in Mecp2-deficient mice and their respective controls, great care was taken at all stages to perform all steps of fixation, freezing, cutting, staining and scanning of the images under the same conditions, using the same solutions and timing, and by alternating tissues coming from the two genotypes. The fluorescence intensity was carefully selected in order to avoid reaching saturation. Densitometric analysis of the staining level was performed on 8-bit images using ImageJ software. The integrated density was calculated as the sum of the values of the pixels region of interest, i.e. in the nuclei as defined by DAPI staining. We analyzed pictures at high magnification (63×) and for each section stained by either αe1, αe2 or Abcam 2828, we measured the Mecp2 staining level of at least 50 DAPI-positive cells. Immunoquantifications were always performed by successively alternating the wild-type and the Mecp2-deficient samples.

## Results and discussion

### Peptide design and rabbit immunization

MeCP2_e1 and MeCP2_e2 differ only by short stretches in their N-terminal regions, which makes it challenging to define isoform-specific peptides that will be long enough to serve as useful immunogens ( Figure 1). In addition, to obtain reagents that would be useful both in mouse and human samples, we had to design and synthesize two peptides that matched both the human and mouse sequences. The first sequence finally chosen corresponded to amino acids 14–24 of human MeCP2_e1 (C-GGGEEERLEEK) and the second to amino acids 1–12 of human MeCP2_e2 (MVAGMLGLREEK-C). Although these peptides do carry the same C-terminal sequence of the three amino acids EEK, we felt that this was very unlikely to lead to cross reactivity since it is commonly accepted that the minimal size for a peptide as an antibody epitope is five amino acids. Cysteine residues were added to the N-terminus of the e1 peptide and to the C-terminus of the e2 peptide for coupling to carrier proteins for immunizations or resins for purification. Rabbits were immunized as described in materials and methods and sera were harvested by terminal exsanguination 9 days after the final injection. Antibodies were affinity purified on columns coupled to their respective peptides, and yielded a total of 2 mg for the αe1 antibody and 3 and 5 mg for the two αe2 antibodies respectively.

In addition, we also generated αe1+e2, a polyclonal antibody reacting against a peptide present in both isoforms of human and mouse MeCP2, as described previously by others ^18^ and ourselves ^19^. This peptide corresponds to amino acids 467–480 of human MeCP2_e1, and 479–492 of human MeCP2_e2.

Finally, we also purchased the commercial antibody Abcam2828, which has been raised against the first 15 amino acids of MeCP2_e2. Since the immunogenic peptide used overlaps only by 6 amino acids with the MeCP2_e1, this antibody would be expected to recognize MeCP2_e2 preferentially, but we feared that it may show some cross reactivity against MeCP2_e1.

### Antibody characterization

**Figure 2.**
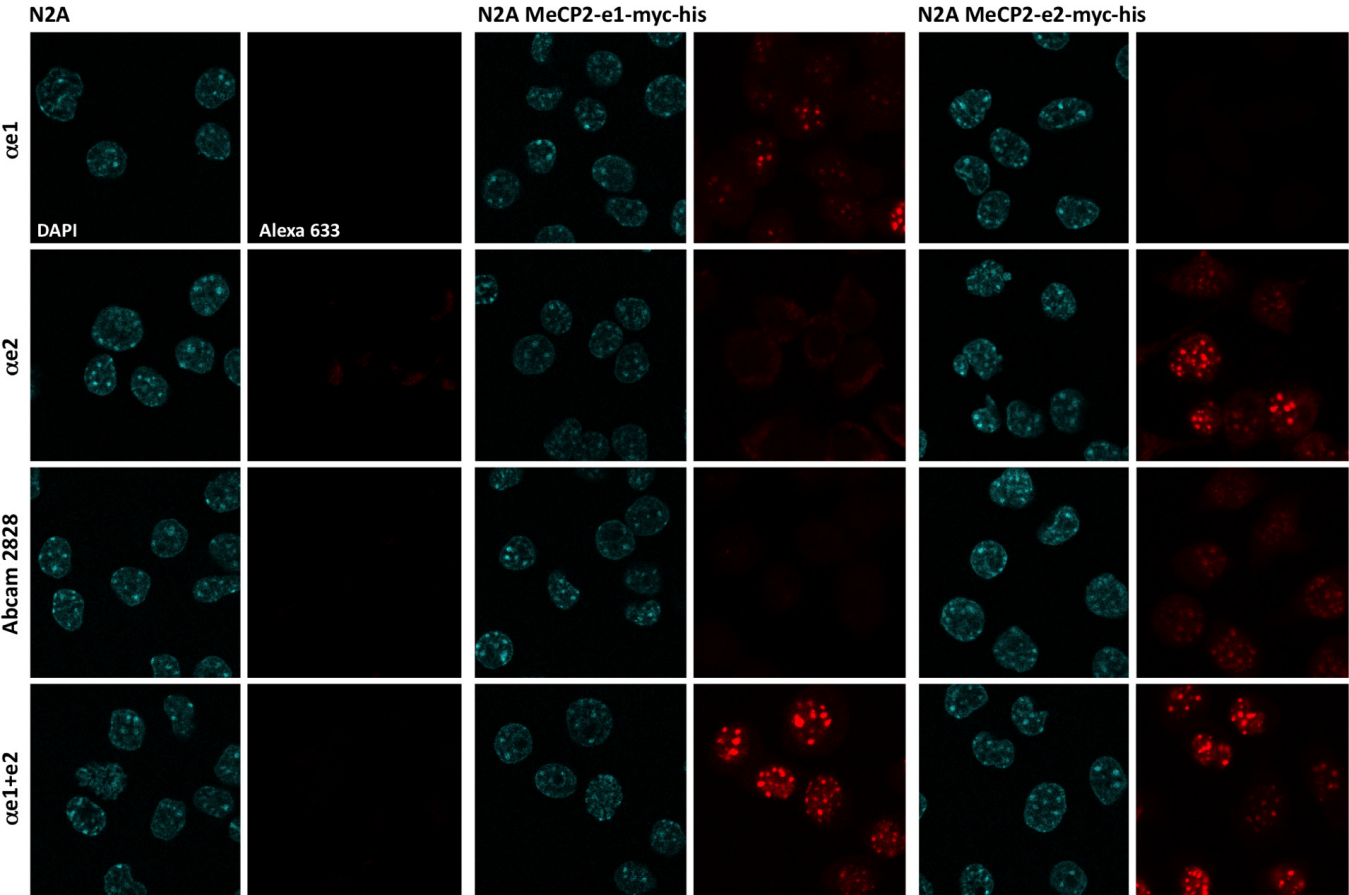
Characterization of the antibodies by immunofluorescence. N2A cells (left columns), or stable N2A transfectants expressing either hMeCP2_e1-myc-his (middle columns) or hMeCP2_e2-myc-his (right columns) were plated on coverslips and labeled with the following antibodies: αe1 (first line), αe2 (second line), Abcam2828 (third line) and αe1+e2 (bottom line), followed by anti-rabbit Alexa633 secondary antibody. Slides were mounted with DAPI-containing medium, and observed with a 40x oil immersion objective. Similar results were obtained in at least five other experiments.

To test the specificity of the antibodies, we used transfected clones of the mouse neuroblastoma cell line N2A which stably express either MeCP2_e1 or MeCP2_e2 (see materials and methods). Despite the neuronal lineage of the N2A cells, it is worth emphasizing that undifferentiated N2A cells do not express any detectable level of endogenous MeCP2 ^23^. As can be seen in Figure 2, after staining with the αe1+e2 antibody, both isoforms were expressed at similar levels in their respective clones, and, as expected, distributed similarly in nuclei, leading to labeling that co-localized closely with heterochromatin, as revealed by DAPI staining.

**Figure S1.**
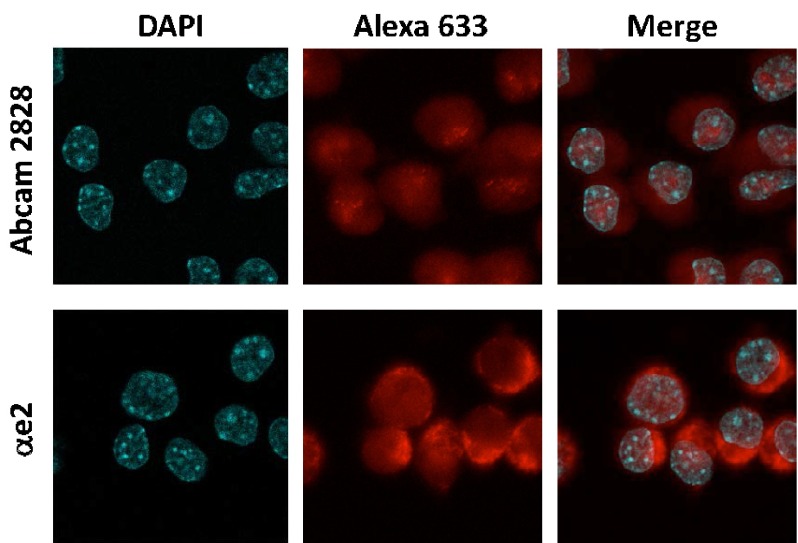
Overexposure of the staining of untransfected N2A cells with αe2 and Abcam2828 (negative controls of Figure 2) reveals that faint staining patterns do not overlap with heterochromatin. For αe2, the signals are mostly cytoplasmic, and for Abcam2828, they are intra-nuclear, but overlap with regions that are less intensely stained with DAPI, and thus more likely correspond to nucleoli than to heterochromatin.

For the αe1 antibody, we found that staining was highly specific of the MeCP2_e1 isoform, with no staining detectable either in the MeCP2_e2 transfectants, or in untransfected N2A cells. Conversely, with the αe2 and Abcam2828 antibodies, we found that the staining obtained was highly specific of the MeCP2_e2 isoform and absolutely no staining was detectable at the level of the heterochromatin in the MeCP2_e1 transfectants. On the other hand, both antibodies gave some background staining on untransfected N2A, but with different and remarkably reproducible patterns: the αe2 antibody led to cytoplasmic background signals, whilst the Abcam2828 antibody gave weak signals that were intra-nuclear, but that were more nucleoplasmic rather than overlapping with heterochromatin ( Figure 2 and Figure S1). It is clear, however, that none of these signals were due to MeCP2 since untransfected N2A cells did not express any detectable levels of MeCP2 with the αe1+e2 antibody ( Figure 2). Quite remarkably, we noticed that the levels of cytoplasmic staining with the αe2 antibody appeared much more intense when there were no cells expressing MeCP2_e2. This suggests that, in the αe2 polyclonal mix, some antibodies that bind with high affinity to the N-terminal portion of MeCP2 may actually cross react with some other cytoplasmic component(s), but with lower affinity. Blast searches of mammalian protein sequences with the sequence of the peptide used for immunizing the rabbits identified proteins of the plectin family as prime candidates since those have cytoplasmic distributions, and many start with MVAGML, the same six amino acids as found in the MeCP2_e2 peptide used for immunizations ( Figure 1B).

### Western blot analyses

**Figure 3.**
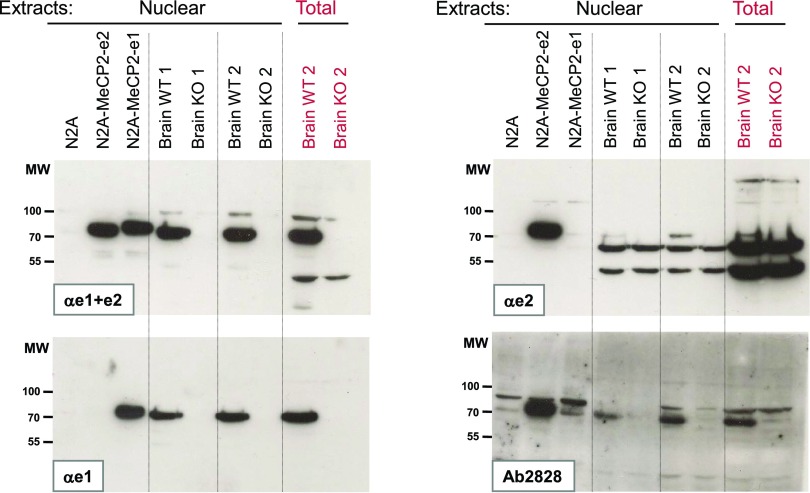
Western blot analysis of MeCP2_e1 and MeCP2_e2 proteins. As described in materials and methods, protein extracts were prepared from N2A cells or from stable N2A transfectants expressing either hMeCP2_e1-myc-his or hMeCP2_e2-myc-his, as well as from brains of two C57 male MeCP2 KO mice, and from their two respective WT littermates. Four nitrocellulose membranes were prepared in parallel from the same extracts, stained respectively with the four antibodies αe1+e2, αe1, αe2 and Abcam2828, and revealed with chemiluminescence. All blots were exposed on the same piece of film for 5 minutes. Similar results were obtained in at least three other experiments.

To characterize the specificity of these antibodies further, we next used Western blot analysis of nuclear and total extracts prepared from the same transfected N2A cells, as well as extracts from either WT or MeCP2 KO mouse brains ( Figure 3).

Staining with the αe1+e2 antibody confirmed the observation made by immunofluorescence that untransfected N2A cells expressed absolutely no detectable endogenous MeCP2, and that both sets of transfectants were expressing similar levels of either MeCP2_e1 or MeCP2_e2. In all experiments, and in agreement with previous reports ^5, 10, 18, 25, 26^, the apparent electrophoretic mobility of the MeCP2 proteins was around 70 KDa, slightly slower than would be expected from the predicted 52 KDa molecular weight. As expected, the e1 isoform, which is 11 amino acids longer, was also found to migrate slightly more slowly than e2. Although the mouse MeCP2_e1 is predicted to be longer than the human form by three amino acids, the slightly slower electrophoretic mobility seen for the human MeCP2 proteins expressed by transfected N2A cells compared to the ones expressed in mouse brains can be explained by the myc-his C-terminal tag carried by this protein, which amounts to slightly more than 20 amino acids ^9^.

Staining with the αe1 and αe2 antibodies confirmed their specificity for their respective isoform, with each revealing only the band corresponding to the MeCP2 isoform it was raised against. For the Abcam2828 antibody, although the signals obtained were clearly almost exclusively detected in the lane containing MeCP2_e2, we could not rule out that this antibody may cross react weakly on MeCP2_e1. Indeed, this antibody shows nonspecific cross-reactivity against two bands which migrate just above and just below the level where MeCP2 is expected, but, in the case shown here, there also was a slightly stronger signal in between these two bands in the N2A-MeCP2_e1 lane than in the N2A control lane. Having repeated this experiment at least four times, we have, however, found it very difficult to document this cross-reactivity reproducibly, with the signal being sometimes undetectable, and sometimes even more prominent than the example shown here.

If we now consider the results obtained with cellular brain extracts of MeCP2-KO and WT mice, we can first see that both αe1+e2 and αe1 efficiently detected a specific signal for MeCP2 in the WT and not in the KO brain extracts. For αe2, although there was a specific signal of the expected size, it was much weaker than the signal obtained with αe1. In fact, in a first set of experiments, we had initially failed to detect this band, and it is only by using very optimized protocols and very sensitive photographic films that we have managed to detect this band in brain extracts reliably.

Another important factor for the detection of this faint band was to use electrophoretic runs that led to good resolution around 50 kDa to separate the MeCP2 signal from the non specific bands running just below. Those two prominent bands, which were not present in the N2A extracts, are clearly non-specific since they are equally present in WT and KO extracts. Since they are also weaker in nuclear than in total cell extracts, they probably correspond to a cytoplasmic protein, possibly of the plectin family alluded to earlier.

With the Abcam2828 antibody, the specific signals corresponding to MeCP2 were much easier to detect than with the αe2 antibody. Since the Abcam2828 antibody may cross-react weakly on MeCP2_e1, the possibility remains that it is not our αe2 antibody which works less well than Abcam2828, but that the stronger signal obtained with the latter may be due to the sum of the detection of the two isoforms.

Altogether, these observations support the already established notion that the e1 isoform is expressed at higher levels in the brain than the e2 isoform.

### Immunostaining of brain sections

**Figure 4.**
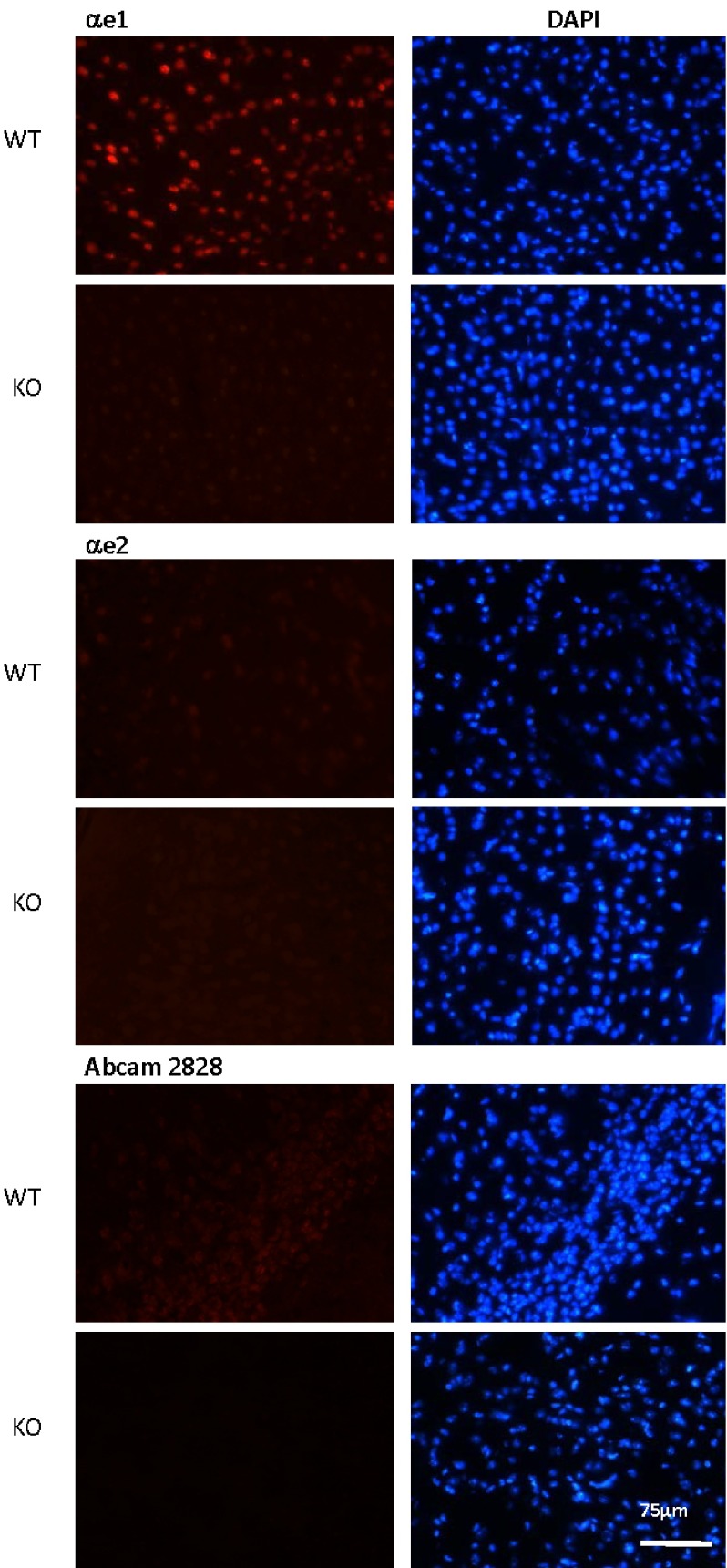
Staining of the lateral posterior thalamic nucleus of WT and MeCP2 KO mice with isoform-specific antibodies. 20 μm brain sections from a C57 male MeCP2 KO mouse, and from a WT littermate, were prepared as described in materials and methods. These sections were stained with αe1 (top section), αe2 (middle section) and Abcam2828 (bottom section), followed by anti-rabbit Alexa 546 secondary antibody, and mounted in DAPI-containing mounting medium.

**Figure S2.**
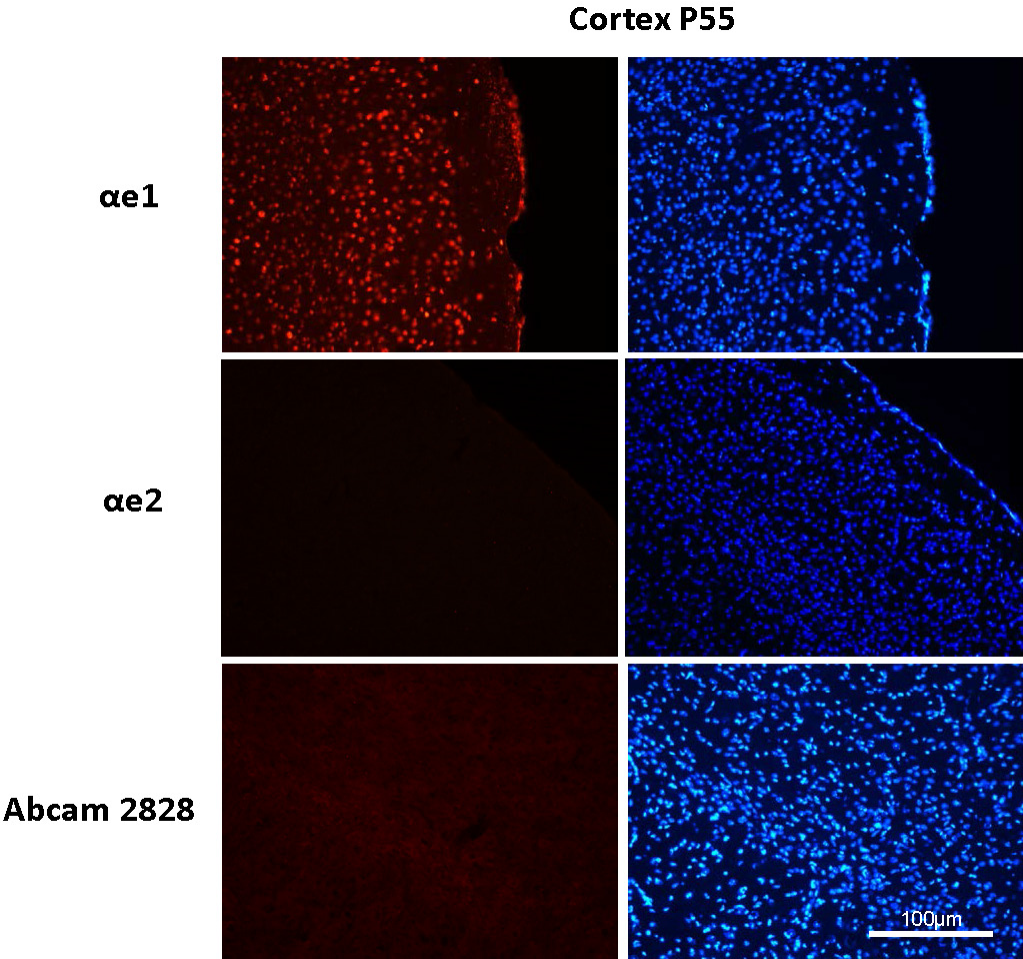
Staining of the cortex of a P55 WT mouse with isoform-specific antibodies. 20 μm brain sections from a C57 male MeCP2 WT mouse were prepared as described in materials and methods. These sections were stained with αe1 (top section), αe2 (middle section) and Abcam2828 (bottom section), followed by anti-rabbit Alexa 546 secondary antibody, and mounted in DAPI-containing mounting medium.

**Figure S4.**
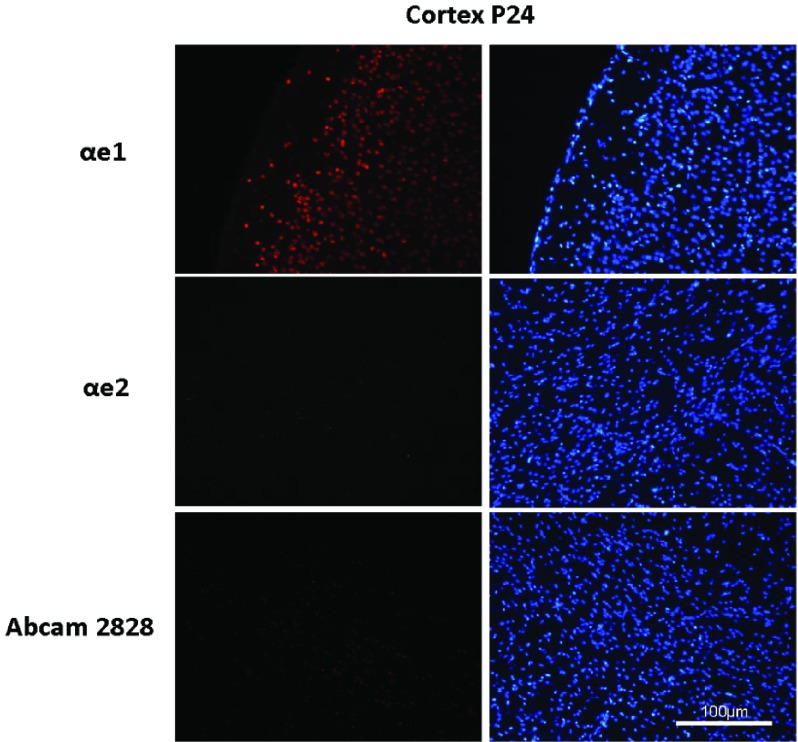
Staining of the cortex of a P24 WT mouse with isoform-specific antibodies. 20 μm brain sections from a C57 male MeCP2 WT mouse were prepared as described in materials and methods. These sections were stained with αe1 (top section), αe2 (middle section) and Abcam2828 (bottom section), followed by anti-rabbit Alexa 546 secondary antibody, and mounted in DAPI-containing mounting medium.

Next, we prepared tissue sections from different regions of the brains of normal C57BL/6 and MeCP2 KO mice. Figure 4 shows examples of the results obtained on lateral posterior thalamic nucleus regions of 55 days old mice. Similar results were obtained with several sections, and on sections of other brain areas such as the cortex and the Substantia Nigra reticulata, and on the cortex of 24 days old mice (see Figure S2– Figure S4). Using the αe1 antibody, we confirmed the absence of labeling on KO tissues while the staining of WT samples revealed the expected nuclear punctuated labeling which co-localized perfectly with the DAPI signals. By contrast, with the αe2 or the Abcam2828 antibodies, we only obtained very faint levels of nuclear staining, which appeared slightly stronger for the Abcam2828 antibody, but were in both cases too weak to be able to discern whether they co-localized with heterochromatin. Although it only provides indicative values, it is possible to use the ImageJ software to quantify the signals obtained by immunofluorescence (see materials and methods). By doing this specifically in nuclei, we found WT/KO signal ratios of about 30 for the αe1 antibody, but only 1,3 for αe2 and 1,8 for Abcam2828, thus bringing further support to the notion that it is mostly the e1 isoform which is expressed in the brain, whilst e2 is only present in trace amounts.

## Discussion

We have succeeded in generating rabbit polyclonal antibodies directed against each of the two MeCP2 isoforms, MeCP2_e1 and MeCP2_e2, and also compared the latter to the commercially available antibody Abcam2828. Whilst each of the two antibodies we have generated were found to be clearly specific of their respective isoform, and suitable for using by Western blot and immunofluorescence, we could not rule out that Abcam2828 did not cross react slightly on MeCP2_e1. Hence, the stronger signals obtained with Abcam2828 on immunostaining brain sections as well as on Western blots with brain extracts could either be due to a better reactivity of this antibody on MeCP2_e2 when it is expressed *in vivo*, or to a weak cross reaction on MeCP2_e1. At any rate, even if the stronger signals obtained with Abcam2828 were due to a better reactivity with MeCP2_e2, our data strongly support the already established views that the MeCP2_e1 isoform is not only the most abundant isoform in the organism, but also the one involved in the physiopathology of RTT ^15^.

This raises the question of why the e2 exon should be conserved in so many, if not all, placental mammals. One possibility is that e2 could be predominantly expressed in other tissues outside the brain. In preliminary experiments, however, we have found a similar predominance of the e1 isoform in lung, heart, liver, spleen and thymus, without managing to detect any significant levels of the e2 protein by Western blot in any of those tissues (unpublished observation). It may be, however, that we did not look in the right place or at the right time. Indeed, very recently published works have suggested that the e2 isoform may play important roles for embryo viability and embryogenesis ^15^, as well as in regulating apoptosis in post-mitotic neurons ^27^. In future experiments, it will thus be interesting to use the αe2 antibody to investigate whether the expression of the e2 isoform can be detected during embryogenesis, or in the brain parenchyma under conditions where post-mitotic neurons undergo apoptosis.

Another intriguing observation in our results concerns the differences seen between the reactivities of the αe2 and Abcam2828 antibodies in immunofluorescence on cells in culture and on brain sections. Indeed, while both antibodies stain N2A cells expressing the e2 isoform with high efficiency, Abcam2828 resulted in noticeably stronger signals on brain sections than the αe2 antibody, albeit still much lower than those obtained with the e1-specific or the pan-reactive reagents. Whilst this may be due to better reactivity of the Abcam2828 antibody on the e2 protein when it is expressed *in vivo*, the possibility remains that this stronger signal may be due to a cross reactivity of that antibody on the e1 isoform since it is expressed at very high levels in the CNS. Although Abcam2828 showed no detectable cross reactivity on the e1 isoform expressed in N2A cells by immunofluorescence, this may be due to the conformation of the e1 isoform not being exactly the same in transfected N2A cells and in mature neurons. It could thus be that, when it is expressed in mature neurons, the e1 isoform can adopt a conformation which is more prone to cross react with Abcam2828, as may well be the case in the context of Western blots. An alternative explanation would be that the e2 isoform is found in a different conformation when expressed in transfected N2A cells or *in vivo*, and that the latter is recognized less efficiently by our αe2 antibody than by Abcam2828. In future experiments, it should be possible to use transgenic mice in which either one or the other of the MeCP2 isoforms has been specifically knocked out ^13, 15^ to discriminate between these two possibilities.

Of note, a very recent report by the group of M. Rastegar has described the generation of an e1-specific chicken polyclonal antibody raised against the very same e1 peptide which we used to immunize rabbits ^28^. Comfortingly, the results we obtained with αe1 are in complete agreement with those in that report, i.e. absolute specificity for the e1 isoform. On tissue slices, both antibodies revealed the same patterns of expression, i.e. strongest in cortical neurons, and overlapping perfectly with the patterns obtained with antibodies that do not discriminate between the two isoforms. While it is somewhat more practical to have reagents raised in the same species to compare the levels of expression of each of the isoforms, having access to reagents raised in different species, such as rabbits and chickens in this case, could prove very useful to combine the two types of reagents for double staining experiments, to monitor expression of the two isoforms in parallel in the same tissue sections.

## Figures and Tables

**Figure S3.**
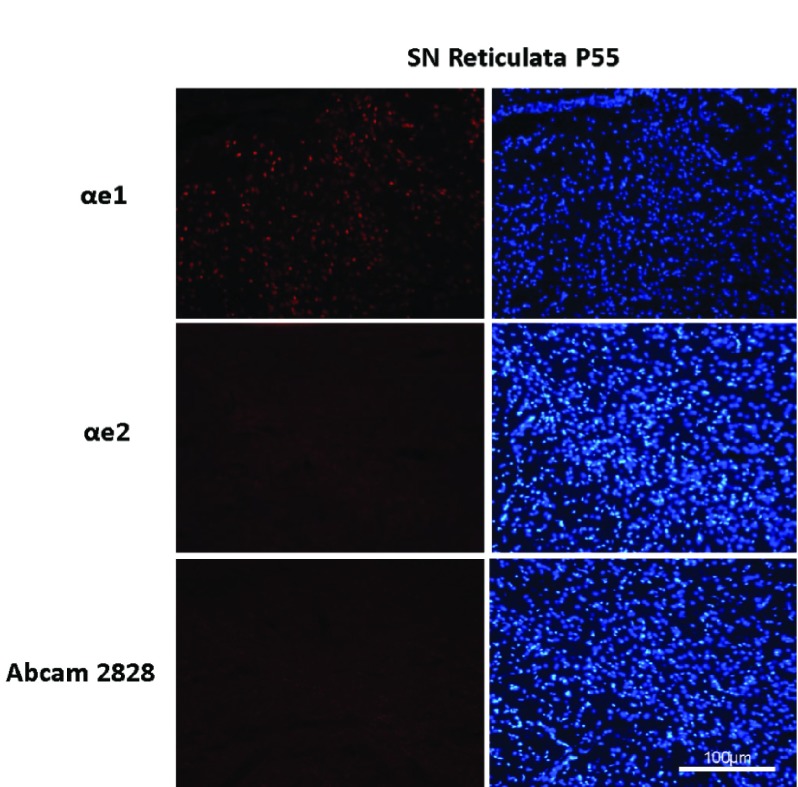
Staining of the substantia nigra reticulata of a P55 WT mouse with isoform-specific antibodies. 20 μm brain sections from a C57 male MeCP2 WT mouse were prepared as described in materials and methods. These sections were stained with αe1 (top section), αe2 (middle section) and Abcam2828 (bottom section), followed by anti-rabbit Alexa 546 secondary antibody, and mounted in DAPI-containing mounting medium.
