# Supplementary material for: Isoform-specific anti-MeCP2 antibodies confirm that expression of the e1 isoform strongly predominates in the brain
Source: F1000Res. 2013 Oct 4;2:204. [Version 1] doi: 10.12688/f1000research.2-204.v1 (PMC3892919; doi:10.12688/f1000research.2-204.v1)
Supplement: Peer review history

# Peer review: Referee response for version 1

**Published:** 2013-11-14

**Authors:** Mike Johnston

**Affiliations:** 1 Division of Neurology & Developmental Medicine, Kennedy Krieger Institute, Baltimore, USA

This interesting paper provides new evidence that the e2 isoform is absent in brain and it is consistent with the earlier paper by Itoh *et al*., 2012 demonstrating that selective deletion of MeCP2_e2 does not result in RTT-associated neurological phenotypes but confers a survival disadvantage to embryos carrying a MeCP2_e2 null allele of maternal origin.

I have read this submission. I believe that I have an appropriate level of expertise to confirm that it is of an acceptable scientific standard.

# Peer review: Referee response for version 1

**Published:** 2013-11-13

**Authors:** Jean-François Brunet

**Affiliations:** 1 Département de Biologie, Institut de Biologie de l'Ecole normale supérieure, Paris, 75005, France

This paper, backed by very convincing and carefully discussed images of immunofluorescence stains and Western blots, describes novel antibodies specific for each of the two MeCP2 isoforms, useful tools no doubt, for the Rett syndrome community. These antibodies allow the authors to conclude, in line with many other clearly summarized previous findings, that one isoform (e1) massively dominates in the adult brain. The text could be shortened a bit.

I have read this submission. I believe that I have an appropriate level of expertise to confirm that it is of an acceptable scientific standard.

# Peer review: Referee response for version 1

**Published:** 2013-10-23

**Authors:** Mario Costa

**Affiliations:** 1 Institute of Neuroscience, National Research Council, Italy, Pisa, Italy

This is a small but interesting paper in which the authors describe the generation of two new and more specific anti-MeCP2 antibodies recognizing the Mecp2 isoforms (Mecp2_e1, and Mecp2_e2). In addition the authors, by using these new tools, are able to revisit and confirm the recent data indicating that MeCP2_e2 is dispensable for healthy brain function, and that it may be involved in the regulation of neuronal apoptosis and embryonic development.

The data and the ideas herein presented are clear and, as also discussed by the authors, there is a growing interest in understanding the functional relationship between the two isoforms of Mecp2. The approach in the antibody preparation is classical, the techniques are well accepted, the data analysis are rigorous and the results are convincing.

Intriguingly, ERK 1/2 (Extracellular Regulated Kinase 1 and 2) like MeCP2 (e1 and e2) presents two different isoforms (in this case originated from two different genes) and the ERK1 isoform posses an N terminal basically identical to that of MeCP2. This data, together with the highly conserved sequence in MeCP2s and ERKs reinforce the idea that this kind of N-terminal could be involved in important biological functions. I think that further speculation on this topic could add additional value to the manuscript.

I have read this submission. I believe that I have an appropriate level of expertise to confirm that it is of an acceptable scientific standard.
